# Synthetic Microbial Communities Enhance Artificial Cyanobacterial Crusts Formation via Spatiotemporal Synergy

**DOI:** 10.3390/microorganisms14010243

**Published:** 2026-01-21

**Authors:** Qi Li, Pingting Zhu, Guoxia Tian, Qingliang Cui, Pengyu Zhang, Lingyan Dong, Chensi Min, Linchuan Fang

**Affiliations:** Key Laboratory of Green Utilization of Critical Non-Metallic Mineral Resources, Ministry of Education, Wuhan University of Technology, Wuhan 430070, China; liqi1689@163.com (Q.L.); zpt123abc@163.com (P.Z.);

**Keywords:** artificial cyanobacterial crusts, synthetic microbial communities, soil nutrient dynamics, microbial biomass

## Abstract

Artificial cyanobacterial crusts (ACCs) are a potentially effective biological strategy for combating desertification. However, while functional microorganisms influence ACCs formation efficiency, research on their role is limited, and their underlying promotion mechanisms remain unclear. Here, we investigated the effects of three functional synthetic microbial communities (SynComs), each dominated by microorganisms specialized in exopolysaccharide (EPS) production (3 strains), siderophore production (3 strains), or nitrogen fixation (4 strains), on ACCs formation following inoculation with *Microcoleus vaginatus*. This study was carried out in a controlled laboratory setting with a 12 h light/dark cycle and a light intensity of 2400–2700 lux. Following a 24-day cultivation period, EPS-producing or nitrogen-fixing SynComs significantly increased the chlorophyll-a content by 16.0–16.3%. Except for the nitrogen-fixing bacteria treatment, other SynComs enhanced the soil organic matter content of ACCs by 9.1% to 27.3%. The content of EPS was significantly improved by all three SynComs by 14.1~19.2%. Urease activity rose by 6.7% when siderophore-producing bacteria were added. The impacts of SynComs on ammonium nitrogen (NH_4_^+^-N) showed different temporal dynamics: nitrogen-fixing SynComs significantly increased NH_4_^+^-N early (≤10 days), while EPS-producing and siderophore-producing SynComs enhanced accumulation later (17–24 days). SynComs inoculation markedly accelerated cyanobacterial and general microbial colonization and growth. In comparison to day 0, the 16S rRNA gene copy number of ACCs increased by 24.1% and 43.0%, respectively, in the EPS-producing and nitrogen-fixing SynComs. Additionally, correlation analysis showed that SynComs transformed the weak correlations in the control into a strong positive correlation between NH_4_^+^-N and both Chl-a and microbial biomass. Our findings demonstrate SynComs, particularly the EPS-producing or nitrogen-fixing SynComs, enhance ACCs formation through elucidated mechanisms, providing a theoretical basis for optimizing ACCs-based desertification control strategies.

## 1. Introduction

Land desertification represents a critical threat to global ecosystem stability and sustainable development. This process depletes nutrients, damages soil structure, and drives vegetation deterioration, ultimately causing severe productivity decline [1,2]. Biological soil crusts (BSCs) are one of the best biological barriers for preventing desertification. These complex, cohesive biofilms formed on soil surfaces, beginning with pioneer cyanobacteria colonization in desert environments, followed by successive establishment of other microorganisms such as bacteria, lichens, and mosses [3,4]. These communities secrete exopolysaccharides (EPS), binding soil particles into a thin yet resilient biological layer. BSCs are essential for preserving the integrity of desert ecosystems because of their many functions [5], which include stabilizing sandy soils, reducing wind and water erosion [6,7], controlling regional hydrological cycles (e.g., enhancing infiltration and reducing evaporation) [8], and driving biogeochemical cycling of carbon, nitrogen, and phosphorus [9,10,11,12]. Despite this ecological significance, natural BSCs development faces dual constraints: extremely slow recovery (>5 years to form functional communities) [13] and accelerated degradation due to climate change and human disturbances [14,15,16]. These intertwined challenges severely impede desert ecosystem self-recovery.

Artificial cyanobacterial crusts (ACCs) have become a promising accelerated technology for desertification prevention and ecological restoration in order to overcome the sluggish production rate of natural biocrusts [17,18,19]. This approach involves selecting dominant cyanobacterial species, large-scale cultivation, and field inoculation [20]. Its success hinges on the effective colonization and growth of cyanobacteria on barren sandy substrates. As a result, efforts have concentrated on enhancing the soil microenvironment through mulch film coverage, straw checkerboard installation, higher inoculation density, and sporadic watering [19,21,22,23]. Although these chemical and physical changes can accelerate cyanobacterial colonization and momentarily increase the availability of nutrients in the soil, they are unable to completely take advantage of the possible synergistic interactions inherent within soil microbial communities. According to recent research, non-cyanobacterial microorganisms in biocrusts quickly revive and start anabolic metabolism within minutes after moisture pulses [24]. This implies microbial community synergism may represent a fundamental biological mechanism underpinning biocrust formation and stabilization. Therefore, it is crucial to create more efficient and long-lasting solutions for restoring desert soil that make use of functional microorganisms.

Synthetic microbial communities (SynComs), constructed by directionally assembling functional microorganisms, provide novel approaches for modulating soil ecological functions. SynComs have shown promise in restoring soil, especially through improved nutrient cycling and stress tolerance [25,26]. SynComs not only directly enhance soil fertility through their own carbon and nitrogen fixation activities [27,28], but also activate inert nutrients such as phosphorus, potassium, and iron in soil minerals by secreting metabolites like organic acids and siderophores [29,30,31]. Metabolic cross-feeding among members strengthens their environmental adaptability [32,33]. Moreover, SynComs can further improve soil microbial diversity and ecosystem stability by introducing new functional metabolisms or stimulating the growth of indigenous microorganisms [34,35,36]. Researchers inoculated EPS-producing bacteria isolated from natural crusts onto the surface of sandy soil, resulting in a significant increase in crust thickness and organic matter transformation efficiency within 30 days [37,38], underscoring the potential of functional microorganisms in BSCs formation. Although natural BSCs contain a variety of functional microbial groups, it is yet unclear which synergistic combinations maximize their combined benefit. Notably, iron and nitrogen are known to be essential components in the nutrient cycling of desert ecosystems. Nitrogen, often the primary limiting nutrient in arid regions, depends heavily on microbial immobilization as a major input pathway [4,39]. Iron is essential for core microbial metabolic processes, such as photosynthesis, nitrogen fixation, and organic matter degradation [40]. Therefore, the construction of SynComs capable of nitrogen fixation, siderophore production, and EPS synthesis is expected to significantly improve the formation rate and ecological functions of ACCs.

Thus, our study isolated EPS-producing, siderophore-producing, and nitrogen-fixing bacteria from natural cyanobacterial crusts. SynComs were constructed based on growth activity and metabolic function compatibility. ACCs cultivation experiments were then carried out to examine dynamic changes in crusts physicochemical properties and microbial biomass. Our objectives were to (i) quantify the promoting effect of SynComs on ACCs formation; (ii) determine functional synergies among SynCom constituents during crust development; and (iii) elucidate the mechanisms by which the SynComs facilitates ACCs formation. These discoveries support the development of sustainable soil remediation techniques based on microbial synergy and offer superior microbial germplasm resources for desert ecological restoration.

## 2. Materials and Methods

### 2.1. Isolation and Identification of Functional Strains

**Figure 1:**
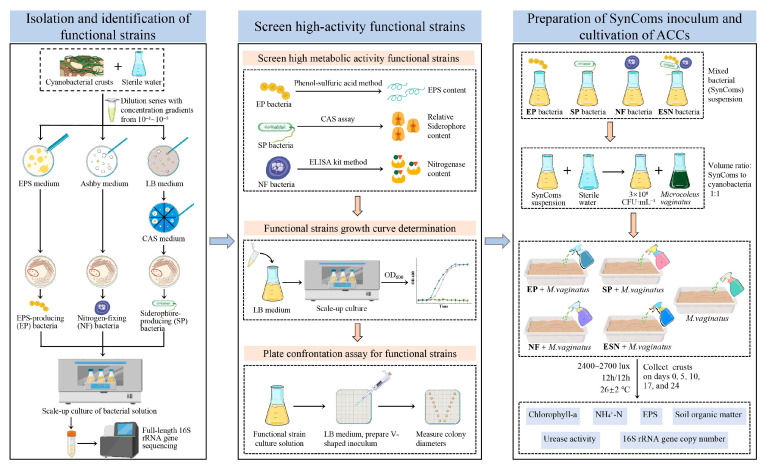
A schematic overview of the experimental workflow.

Natural cyanobacterial crusts were collected in August 2024 from the Shapotou Region of Ningxia Hui Autonomous Region, located on the southeastern edge of the Tengger Desert in China (37°32′ N, 105°02′ E; 1339 m elevation), for the isolation of functional strains. One gram of cyanobacterial crust was added to 10 mL of sterile water and shaken at 150 rpm for 2 h. The suspension was then diluted with sterile water to generate five serial dilutions (10^−1^~10^−5^). Fifty microliters of each dilution was plated onto Luria–Bertani (LB), Ashby’s Nitrogen-free (Ashby), and exopolysaccharide (EPS) solid medium, followed by incubation at 30 °C for 36~48 h. EPS and Ashby solid media were employed for the isolation of EPS-producing and nitrogen-fixing bacteria, respectively. Partition the Chrome azurol S Assay Medium (CAS) solid medium into 8 equal sections. The LB, Ashby, and CAS media were purchased from Qingdao Hi-Tech Industrial Park Hope Bio-Technology Co., Ltd. (Qingdao, China). LB medium: tryptone 10.0 g·L^−1^, yeast extract 5.0 g·L^−1^, NaCl 10.0 g·L^−1^, pH 7.0 ± 0.1 (25 °C). Ashby’s Nitrogen-free medium: mannitol 10.0 g·L^−1^, KH_2_PO_4_ 0.2 g·L^−1^, MgSO_4_ 0.2 g·L^−1^, NaCl 0.2 g·L^−1^, CaCO_3_ 5.0 g·L^−1^, CaSO_4_ 0.1 g·L^−1^, pH 7.0 ± 0.1 (25 °C). EPS medium: yeast extract 5.0 g·L^−1^, peptone 5 g·L^−1^, casein acid hydrolysate 5.0 g·L^−1^, glucose 5 g·L^−1^, soluble starch 5 g·L^−1^, sodium pyruvate 5 g·L^−1^, K_2_HPO_4_ 3 g·L^−1^, MgSO_4_ 0.5 g·L^−1^, pH 7.0–7.5 (25 °C) [37]. Solid medium supplemented with 15 g·L^−1^ agar. CAS solid medium: chrome azurol S 60.5 mg·L^−1^, cetyl trimethyl ammonium bromide 72.9 mg·L^−1^, FeCl_3_·6H_2_O 2.645 mg·L^−1^, NaH_2_PO_4_·2H_2_O 295.25 mg·L^−1^, Na_2_HPO_4_·12H_2_O 1213.5 mg·L^−1^, NH4Cl 125 mg, KH_2_PO_4_ 37.5 mg, NaCl 62.5 mg, agar 9 g·L^−1^, pH 6.8 ± 0.1 (25 °C) (Figure 1).

Vigorously growing colonies from the LB plate were picked and inoculated onto the CAS medium—one colony per section—followed by incubation at 30 °C. Colonies that yellowed the medium were identified as siderophore-producing bacteria. The isolated EPS-producing, siderophore-producing, and nitrogen-fixing bacteria were subcultured in LB medium for proliferation. The expanded cultures were submitted to Wuhan Hecegene Technology Co., Ltd. (Wuhan, China) for full-length 16S rRNA gene sequencing. The obtained full-length 16S rDNA sequences of the strains were aligned against the NCBI GenBank database. The full-length 16s rDNA sequences obtained from the strains were compared against the NCBI GenBank using BLAST+ 2.17.0.

### 2.2. Determination of Metabolic Activity of Functional Strains

The EPS, siderophore, and nitrogenase production capacities of the functional strains were determined using the phenol–sulfuric acid method [41], CAS assay [42], and ELISA kit (Shanghai Baililai Biotechnology Co., Ltd., Shanghai, China), respectively, with triplicate measurements conducted for each strain. For EPS quantification via the phenol–sulfuric acid method, absorbance was measured at 490 nm using a spectrophotometer. The glucose standard curve equation was *Y* = 0.62 *X* + 0.03 (*R*^2^ = 0.999), where *Y* represents the absorbance and *X* corresponds to the glucose concentration (g/L).

A 1 mL aliquot of a 5-day-old seed culture was inoculated into 100 mL of LB liquid medium and incubated at 30 °C with shaking at 150 rpm. Samples were collected every 2 h for optical density (OD) measurement at 600 nm (OD_600_) using a spectrophotometer. Growth curves were constructed by plotting OD_600_ values against culture time (hours). Triplicate measurements were conducted for each sampling point to ensure experimental reproducibility.

### 2.3. Plate Confrontation Assay for Functional Strains

Seven 1 μL aliquots of a pre-cultured bacterial suspension were sequentially inoculated along the two diagonals of a 10 cm × 10 cm square LB solid medium plate, with inoculation points gradually converging into a “V” shape [43]. Plates were sealed with Parafilm and incubated at 30 °C for 24~28 h. Colony diameters were measured using a vernier caliper (resolution: 0.01 mm), and average colony diameters were calculated by excluding the maximum and minimum values from each set of measurements.

### 2.4. Preparation of SynComs Liquid Inoculum and Cultivation of ACCs

Based on the above results, strains with high metabolic activity and without antagonistic interactions were selected to construct a SynComs. Four functional types of SynComs were constructed: (1) EPS-producing bacteria, (2) siderophore-producing bacteria, (3) nitrogen-fixing bacteria, and (4) a SynComs combining all three functions. Prior to inoculation, each strain was cultured individually. At the time of inoculation, the strains were mixed in equal volumes to form a composite suspension. The mixed bacterial suspension was first adjusted to an OD_600_ of 0.4 using sterile water, centrifuged to remove the supernatant, and resuspension in sterile water to a cell density of 3 × 10^8^ CFU·mL^−1^. *Microcoleus vaginatus* was ground and then cultured in BG11 liquid medium for 28 days under the following conditions: light intensity of 2400~2700 lux, light–dark cycle of 12 h/12 h, and temperature of 26 ± 2 °C. The cyanobacteria biomass was adjusted to 2.4 mg·mL^−1^ before inoculation. Each of the four SynComs inoculants was mixed with the cyanobacteria suspension at a 1:1 (*v*/*v*) volume ratio. Each inoculum mixture was adjusted to a final volume of 50 mL, containing a mixed bacterial solution at a concentration of 1.5 × 10^8^ CFU·mL^−1^. Additionally, a cyanobacteria-only control group was set up, where the cyanobacteria suspension was mixed with sterile water in equal volumes. Sand collected from the Tengger Desert was spread uniformly in plastic trays (34.5 cm × 22.0 cm base area) to a thickness of ~1 cm. The inoculum mixtures were evenly sprayed onto the sand surface, with three replicates per treatment. Trays were placed on a light culture rack under the following conditions: culture temperature of 26 ± 2 °C, light intensity of 2400~2700 lux, and a 12 h/12 h light–dark cycle. Distilled water was sprayed onto the trays every other day to maintain soil moisture content at approximately 10%. Crusts samples were collected on days 0, 5, 10, 17, and 24.

### 2.5. Physicochemical Property Determination of ACCs

ACCs were fully ground using mortars and pestles and passed through 80-mesh sieves. One portion was placed in kraft paper envelope, stored in a glass desiccator in the dark for physicochemical analysis, while the other portion was stored at −80 °C for subsequent DNA extraction. Chlorophyll-a (Chl-a), ammonium nitrogen (NH_4_^+^-N), and EPS contents were determined via the ethanol extraction [44], indophenol blue colorimetric [45], and phenol–sulfuric acid method [46], respectively. Urease activity and soil organic matter content were determined using commercial kits (Suzhou Keming Biotechnology Co., Ltd., Suzhou, China) following the manufacturer’s instructions. Three replicates for each sample.

### 2.6. Quantitative Real-Time PCR

Approximately 0.2 g of crusts sample was used for total soil DNA extraction via a column-based method. DNA quality and concentration were assessed using agarose gel electrophoresis and NanoDrop 2000c spectrophotometer (Thermo Fisher Scientific, Waltham, MA, USA), respectively. Absolute bacterial abundance was quantified via qPCR with primers 515F/806R [47]. Each 20 µL reaction system contains 10 μL 2 × T5 Fast qPCR Mix (SYBR Green I, Biosharp, Beijing, China), 0.8 μL of each primer, 1 μL DNA template, and 7.4 μL ddH_2_O. The annealing step was performed at 57 °C for 15 s. The qPCR assay exhibited an amplification efficiency of 94.5%, with a standard curve defined by the equation: *Y* = −3.4625*X* + 42.85 (*R*^2^ = 0.998), where *Y* denotes the cycle threshold (Ct) value and *X* corresponds to the logarithm of the initial template concentration. All the PCRs were conducted in triplicates.

### 2.7. Statistical Analysis

One-way ANOVA was performed using SPSS 24.0 software (SPSS Inc., Chicago, IL, USA) to test for significant differences (*p* < 0.05) in various indicators among different treatment groups. Normality of the data was first assessed using the Shapiro–Wilk test. Upon confirmation of normality, homogeneity of variances was evaluated via Levene’s test. For datasets with homogeneous variances, statistical significance was determined using one-way ANOVA followed by LSD’s HSD post hoc test. In cases of non-homogeneous variances, Welch’s ANOVA was employed, with Tamhane’s T2 test used for post hoc comparisons. A bacterial neighbor-joining phylogenetic tree was constructed using MEGA 12 software. Evolutionary distances were calculated using the maximum composite likelihood method prior to tree construction, and bootstrap analysis (1000 replications) was performed to assess confidence in the tree topology.

## 3. Results

### 3.1. Functional Strain Screening and Identification

#### 3.1.1. Screening and Identification of Highly Active Functional Strains

**Figure 2:**
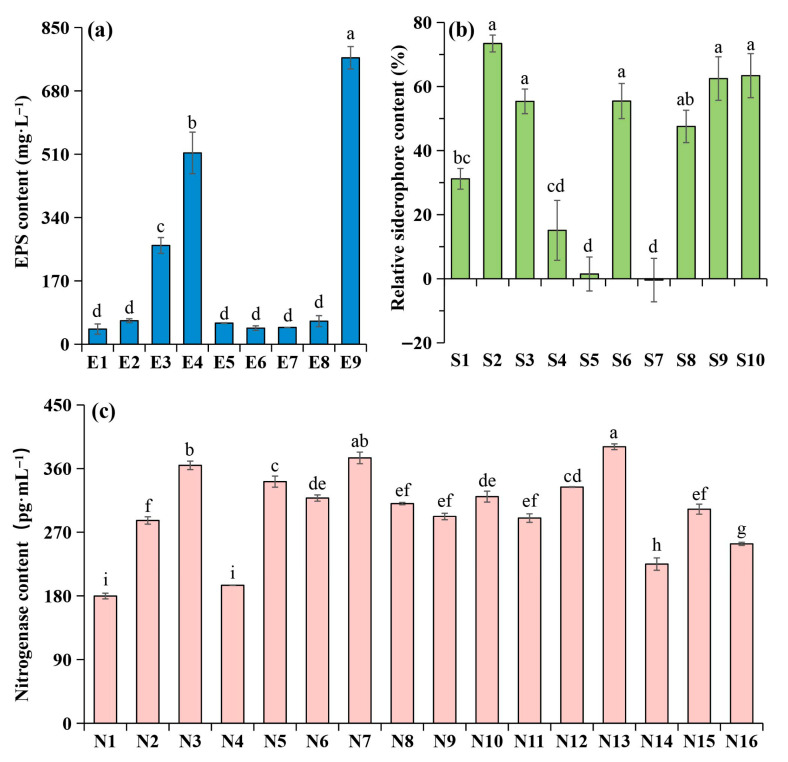
EPS production (**a**), siderophore production (**b**), and nitrogenase content (**c**) in functional strains. Error bars represent the mean ± SD. Different lowercase letters indicate significant differences (LSD’s HSD test; *p* < 0.05) among different strains (n = 3).

A totla of 9 EPS-producing strains, 10 siderophore-producing strains, and 16 nitrogen-fixing strains were isolated and designated as E1–E9, S1–S10, and N1–N16, respectively. Among the EPS-producing strains, strain E9 had the highest EPS yield of 769 mg/L (*p* < 0.001), followed by E4 (514 mg·L^−1^) and E3 (265 mg·L^−1^). The remaining six strains exhibited significantly lower yields, ranging from 40.8 to 63.2 mg/L (Figure 2a). For siderophore production, strains S2, S3, S6, S8, S9, and S10 exhibited significantly higher siderophore activity (*p* < 0.001), with relative contents ranging from 55.4% to 73.4% (Figure 2b). For nitrogenase content, strains N13, N12, N7, N3, and N5 had significantly higher levels (*p* < 0.001), with contents ranging from 334 to 391 pg/mL (Figure 2c). Growth curve analysis showed that strains E3, E4, and E9 entered the logarithmic phase within 8 h (Figure S1a). Strain N7 exhibited a markedly lower OD600 than other nitrogen-fixing strains under the same conditions (Figure S1b). Siderophore-producing strains (S2, S3, S6, S8, S9, and S10) entered the logarithmic phase after 4 h, although S3 and S9 showed significantly lower OD_600_ compared to the other siderophore-producing strains (*p* < 0.01) (Figure S1c). In summary, strains E3, E4, E9, N3, N5, N12, N13, S2, S6, S8, and S10 were selected for subsequent experiments.

**Figure 3:**
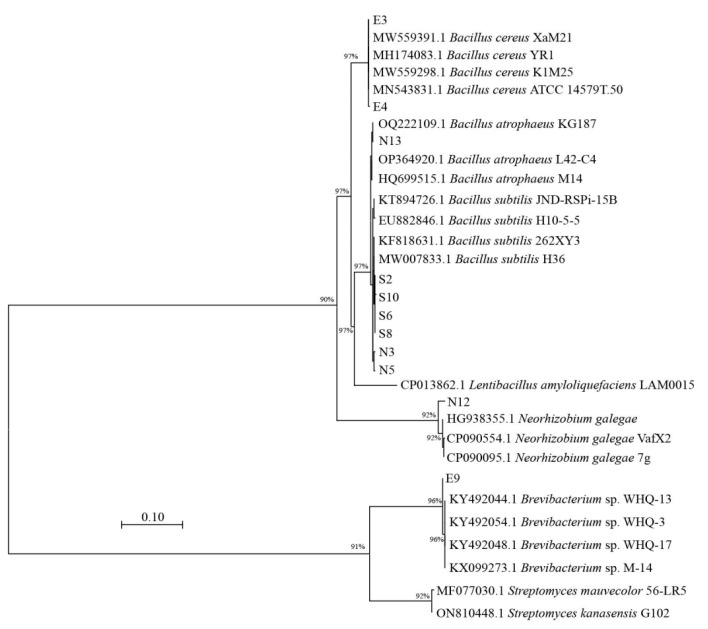
Phylogenetic tree of highly active functional strains constructed based on 16S rRNA gene sequences. Bootstrap values (1000 replicates) are shown at nodes.

To determine the precise taxonomic affiliations of the highly active functional strains, a phylogenetic tree was constructed based on 16S rDNA sequences (Figure 3). Strains E3 and E4 exhibited 97% sequence similarity to *Bacillus cereus*, whereas E9 showed 96% similarity to *Brevibacterium* sp. Four siderophore-producing strains (S2, S6, S8, S10) and two nitrogen-fixing strains (N3, N5) both showed 97% sequence similarity to *Bacillus subtilis*. Strain N13 exhibited 97% sequence similarity to *Bacillus atrophaeus*, whereas strain N12 showed only 92% sequence similarity to *Neorhizobium galegeae*. Therefore, E3, E4, S2, S6, S8, S10, N3, N5, and N13 belong to the genus *Bacillus*, E9 belongs to the genus *Brevibacterium*, and N12 belongs to the family Rhizobiaceae.

#### 3.1.2. Comparison of Inhibition Zone Diameters Among Highly Active Functional Strains

Confrontation assay results for strains within the same functional group are shown in Supplementary Figures S2 and S3. Three EPS-producing strains (E3, E4, E9) exhibited either mutual promotion or weak inhibition, while four nitrogen-fixing strains (N3, N5, N12, N13) showed weak inter-strain inhibition. Among siderophore-producing strains, strain S2 has a significant inhibitory effect against the other strains (S6, S8, S10) (*p* < 0.001), with inhibition rates ranging from 26.6% to 32.1%. Consequently, S2 was excluded from subsequent experiments.

**Figure 4:**
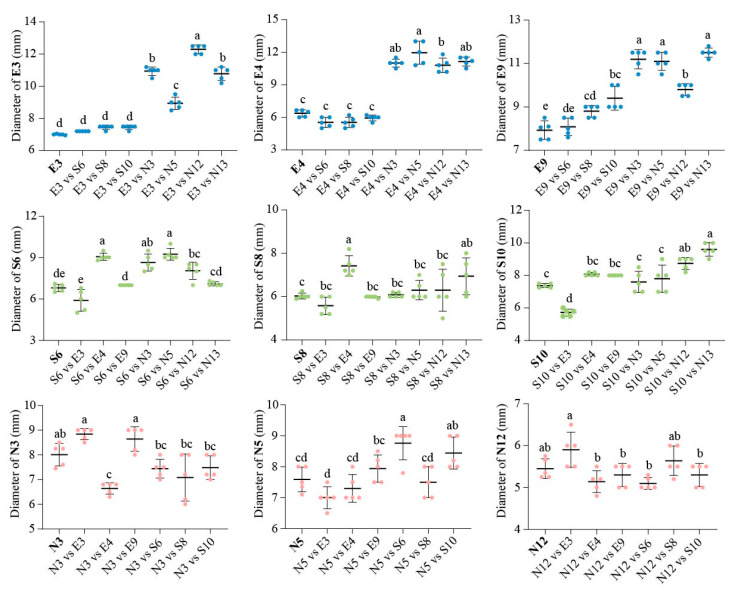
Colony diameters (mm) in plate confrontation assay among different highly active functional strains. Error bars represent the mean ± SD. Different lowercase letters indicate significant differences (LSD’s HSD test; *p* < 0.05) among different strains (n = 5).

Results of the confrontation assay between strains from different functional groups are presented in Figure 4 and Supplementary Figure S4. Four nitrogen-fixing strains significantly promoted the growth of all three EPS-producing strains (*p* < 0.001), demonstrating a strong cross-functional promotional effect. Conversely, the three EPS-producing strains had no significant promotional or inhibitory effects on two nitrogen-fixing strains (N5, N12). For the remaining two nitrogen-fixing strains (N3, N13), E3 and E9 exerted a moderate promotional effect, whereas E4 showed a mild inhibitory effect on their growth. Among the three siderophore-producing strains, S8 and S10 significantly promoted the growth of E9 (*p* < 0.001), whereas the other strain (S6) had no significant effect on E3 and E4. Conversely, E4 and E9 exerted a moderate promotional effect on S6, S8, and S10. For nitrogen-fixing strains, N12 and N13 significantly promoted the growth of all three siderophore-producing strains (*p* < 0.001), whereas N3 and N5 exerted significant promotional effects only on S6 and S8. For the reverse interaction (siderophore-producing strains on nitrogen-fixing strains), S6 and S10 mildly inhibited the growth of N3, N12, and N13 but significantly promoted N5 (*p* < 0.001). Additionally, S8 exerted a significant promotional effect on N13 (*p* < 0.001). Taken together, three EPS-producing strains (E3, E4, E9), three siderophore-producing strains (S6, S8, S10), and four nitrogen-fixing strains (N3, N5, N12, N13) displayed either mutual promotion or merely weak inhibitory interactions, suggesting these ten strains are well-suited for co-inoculation.

### 3.2. Effects of Different Functional SynComs on Physicochemical Properties of ACCs

#### 3.2.1. Chl-a Content Responses to Different Functional SynComs

**Figure 5:**
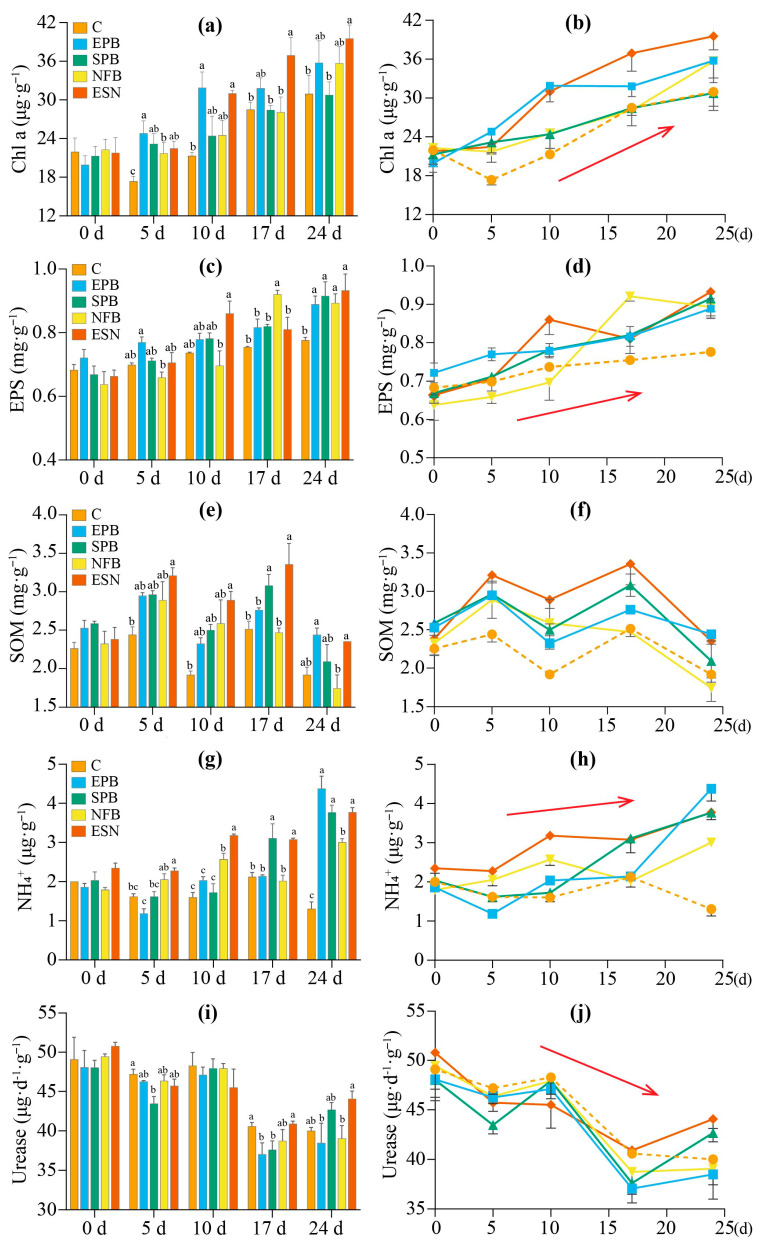
Temporal changes in physicochemical properties of ACCs inoculated with different functional SynComs. (**a**,**b**) Chlorophyll-a (Chl-a). (**c**,**d**) Exopolysaccharide (EPS). (**e**,**f**) Soil organic matter (SOM). (**g**,**h**) Ammonium nitrogen (NH_4_^+^-N). (**i**,**j**) Urease activity. C, *Microcoleus vaginatus* monoculture (control); EPB, EPS-producing bacteria + *M. vaginatus*; SPB, siderophore-producing bacteria + *M. vaginatus*; NFB, nitrogen-fixing bacteria + *M. vaginatus*; ESN, EPS-, siderophore-, and nitrogen-fixing bacteria + *M. vaginatus*. The red arrow denotes the trend of variation. Error bars represent the mean ± SD. Different lowercase letters indicate significant differences (LSD’s HSD test; *p* < 0.05) among different strains (n = 3).

Chl-a content exhibited a significant upward trend across all treatment groups over the 24-day cultivation period (Figure 5a,b). In the early stage (≤10 days), all four SynComs groups showed significantly higher Chl-a content (21.7–31.9 μg·g^−1^) relative to the control group (17.4–21.9 μg·g^−1^) (day 5, *p* = 0.045; day 10, *p* = 0.018). Notably, the EPB treatment achieved the highest Chl-a content on day 5 (24.8 μg·g^−1^) and day 10 (31.9 μg·g^−1^), representing 24.5% and 59.9% increases, respectively. On day 17, the ESN treatment exhibited the highest Chl-a content (36.9 μg·g^−1^), followed by the EPB (31.8 μg·g^−1^) treatment, while the SPB (28.4 μg·g^−1^), NFB (28.1 μg·g^−1^) and control (28.5 μg·g^−1^) groups had relatively lower values. On day 24, significant differences in Chl-a content were detected among treatment groups (*p* = 0.045): the ESN (39.5 μg·g^−1^), EPB (35.8 μg·g^−1^), and NFB (35.7 μg·g^−1^) groups were significantly higher than the SPB (30.8 μg·g^−1^) and control (30.9 μg·g^−1^) groups. Taken together, all four SynComs effectively and significantly increased Chl-a content in ACCs, and the promotional effects of different SynComs on Chl-a exhibited stage-specific patterns.

#### 3.2.2. EPS Content as Affected by Different Functional SynComs

EPS content of all five ACCs exhibited a significant increasing trend over incubation time (Figure 5c,d). On day 0, the EPB treatment had a slightly higher EPS content (0.72 mg·g^−1^) than the other four treatment groups (0.63–0.68 mg·g^−1^), though no significant difference was detected among all groups (*p* = 0.339). By day 5, the EPB treatment maintained the highest EPS content (0.77 mg·g^−1^), while the NFB treatment showed the lowest value (0.66 mg·g^−1^) (*p* = 0.022). This pattern shifted by day 10: the ESN treatment emerged as the top performer (0.86 mg·g^−1^), and the NFB treatment remained significantly lower than other treatments (0.70 mg·g^−1^, *p* = 0.028). However, on day 17, the NFB treatment showed the significantly highest EPS content (0.92 mg·g^−1^, *p* = 0.004), while the other three SynComs treatment groups (EPB, SPB, ESN) displayed slightly higher EPS contents compared to the control group. By day 24, all four SynComs treatment groups exhibited significantly higher EPS contents (0.89–0.93 mg·g^−1^) compared to the control group (0.78 mg·g^−1^, *p* = 0.020). These results indicate that the enhancement of EPS content in ACCs by different SynCom exhibits temporal variability. When cultivation time was ≤10 days, the addition of EPS-producing or nitrogen-fixing bacteria showed a more pronounced promoting effect on EPS accumulation. After 24 days of cultivation, the addition of EPS-producing, siderophore-producing, or nitrogen-fixing bacteria results in similar enhancement of EPS content.

#### 3.2.3. Organic Matter Content Responses to Different Functional SynComs

The soil organic matter (SOM) content in all five ACCs treatments exhibited a fluctuating pattern with an overall downward trend over the experimental period (Figure 5e,f). At the initial inoculation time (day 0), SOM content in the four SynComs (EPB, SPB, NFB, ESN) was modestly higher (2.32–2.59 mg·g^−1^) than that in the control group (2.15 mg·g^−1^). Compared to day 0, SOM content significantly increased in all five treatments on days 5 and 17 (*p* < 0.05), but decreased on days 10 and 24. On days 5 and 10, SOM content in the four SynComs treatment groups was statistically significantly higher than that in the control group (day 5, *p* = 0.021; day 10, *p* = 0.015). By days 17 and 24, all SynComs groups remained higher in SOM content than the control group, except for the NFB group. Notably, the ESN group exhibited significantly higher SOM content than the control from day 5 to 24 (*p* < 0.05), indicating a sustained positive effect of the multi-functional SynComs on SOM accumulation.

#### 3.2.4. Responses of Ammonium Nitrogen Content and Urease Activity to Different Functional SynComs

The NH_4_^+^-N content in the four SynComs groups exhibited a fluctuating but overall increasing trend throughout the experimental period (Figure 5g,h). For the EPB, SPB, and ESN groups, NH_4_^+^-N contents followed a pattern of initial decline (day 0 to day 5) followed by a marked increase, rising from baseline values of 1.86, 2.04, and 2.35 µg·g^−1^ to final concentrations of 4.38, 3.77, and 3.77 µg·g^−1^, respectively. In contrast, the NFB group showed a temporary decrease on day 17 but gradually increased during other stages, eventually reaching 3.01 µg·g^−1^. The control group displayed an overall decreasing trend from 2.00 to 1.31 µg·g^−1^, albeit with a temporary rise on day 17. Groups with significantly higher NH_4_^+^-N content varied across different culture stages. Early stage (days 5–10): The ESN and NFB groups had the highest NH_4_^+^-N contents, with the NFB group showing a pronounced 1.44-fold increase from day 0 to day 10 (*p* < 0.05). This highlights the dominant role of nitrogen-fixing strains in early nitrogen accumulation. Mid-stage (day 17): The EPB and ESN groups emerged as top performers, with the SPB group exhibiting a 1.47-fold higher NH_4_^+^-N content than the control (*p* = 0.003). Late stage (day 24): The EPB, SPB, and ESN groups maintained the highest NH_4_^+^-N contents, with the EPB group showing a 2.35-fold increase from day 0 (*p* < 0.001). In summary, inoculation with nitrogen-fixing bacteria most significantly enhances ammonium nitrogen content when the ACCs cultivation time is ≤10 d. In contrast, during the cultivation period of 17–24 days, the treatments inoculated with EPS-producing bacteria or siderophore-producing bacteria performed better, with ammonium nitrogen content increases 6.02–54.21% higher than those in the nitrogen-fixing bacteria treatment. This dynamic variation trend was consistent with the spatiotemporal variation in EPS content.

In different types of ACCs, urease activity exhibited a significant decreasing trend as cultivation time prolonged (Figure 5i,j). At the initial stage, the urease activity of the five groups ranged from 48.0 to 50.8 μg·d^−1^·g^−1^. After 5 days of cultivation, a mild decrease was detected, followed by a temporary rebound in activity on days 10 and 24. Notably, on day 17, urease activity dropped significantly to 37.1–40.9 μg·d^−1^·g^−1^ (*p* < 0.05). Across all culture stages, the control group consistently maintained significantly higher urease activity compared to other groups (*p* < 0.05). The ESN group exhibited higher urease activity on days 17 and 24, recording values of 40.9 and 44.1 μg·d^−1^·g^−1^, respectively. The SPB group showed a more restricted advantage: it exhibited a significantly higher urease activity exclusively on day 24 (42.7 μg·d^−1^·g^−1^, *p* = 0.022).

#### 3.2.5. 16S rRNA Gene Copy Number Responses to Different Functional SynComs

**Figure 6:**
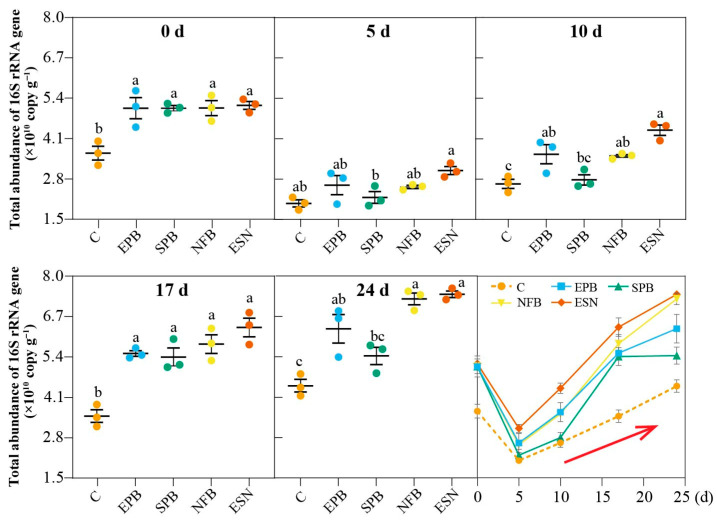
Changes in 16S rRNA gene copy number across ACCs supplemented with different SynComs. C, *Microcoleus vaginatus* monoculture (control); EPB, EPS-producing bacteria + *M. vaginatus*; SPB, siderophore-producing bacteria + *M. vaginatus*; NFB, nitrogen-fixing bacteria + *M. vaginatus*; ESN, EPS-, siderophore-, and nitrogen-fixing bacteria + *M. vaginatus*. The red arrow denotes the trend of variation. Error bars represent the mean ± SD. Different lowercase letters indicate significant differences (LSD’s HSD test; *p* < 0.05) among different strains (n = 3).

At the early stage (0–5 days), the 16S rDNA copy numbers of the five ACCs exhibited a significant decreasing trend, with reductions ranging from 40.6% to 56.6% (Figure 6). Notably, the ESN group showed the least reduction. As cultivation progressed, gene copy numbers in all groups increased gradually. By days 10, 17, and 24, the ESN group consistently exhibited the highest gene copy numbers compared to the other groups (*p* < 0.05), reaching 4.37 × 10^10^, 6.35 × 10^10^, and 7.41 × 10^10^ copies/g soil, respectively. This was followed by the NFB and EPB groups, with copy numbers ranging from 3.54 × 10^10^ to 7.27 × 10^10^ copies/g soil. On day 17, all four SynComs groups (EPB, SPB, NFB, ESN) had significantly higher gene copy numbers than the initial value (*p* < 0.05), whereas the control group did not exhibit a significant increase relative to the initial value until day 24. By day 24, the EPB, SPB, NFB, and ESN groups had increased 2.42-, 2.46-, 2.86-, and 2.41-fold relative to their respective day 5 gene copy numbers, whereas the control group showed a 2.19-fold increase. These results suggest that the addition of SynComs shortened the microbial adaptation period and enhanced microbial colonization within the crusts.

#### 3.2.6. Impact of Different Functional SynComs on the Correlation Between Soil Physicochemical and Biomass

**Figure 7:**
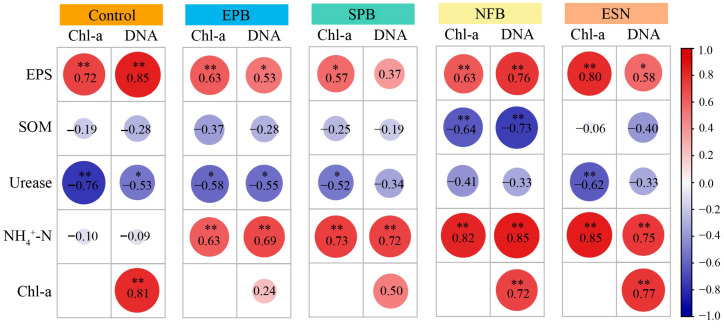
Spearman correlation between physicochemical indices and biomass of different ACCs. Control, *Microcoleus vaginatus* monoculture (control); EPB, EPS-producing bacteria + *M. vaginatus*; SPB, siderophore-producing bacteria + *M. vaginatus*; NFB, nitrogen-fixing bacteria + *M. vaginatus*; ESN, EPS-, siderophore-, and nitrogen-fixing bacteria + *M. vaginatus*. Chl-a, chlorophyll-a; EPS, exopolysaccharide; SOM, soil organic matter; NH_4_^+^-N, ammonium nitrogen; Urease, urease activity. Significance between physicochemical variables is indicated by an asterisk (* represents *p* < 0.05; ** represents *p* < 0.01).

Correlation analysis clarified the key relationships between soil physicochemical variables and biomass indicators (Chl-a, DNA) during artificial cyanobacterial biocrusts formation amended with functional SynComs (Figure 7). NH_4_^+^-N, a key intermediate in organic-inorganic nitrogen conversion, showed markedly different correlations with biomass between control and SynComs groups: no significant correlation was observed in the control (r ≥ −0.10), whereas all four SynComs groups (EPB, SPB, NFB, ESN) exhibited strong positive correlations (*p* < 0.05, r = 0.63–0.85), with the NFB and ESN groups showing the highest coefficients (r ≥ 0.75). Across all groups, Chl-a was positively correlated with DNA, and EPS was also positively correlated with both, but the strongest correlations were in the control group, followed by the ESN and NFB groups. SOM showed a significant negative correlation with Chl-a and DNA only in the NFB group (*p* < 0.01, r = −0.64 to −0.73). In the control, urease activity negatively correlated with Chl-a and DNA (r < −0.50, *p* < 0.05), but this correlation weakened in four SynComs groups, particularly in NFB where no significance was detected (*p* > 0.05). Our findings revealed that functional SynComs altered correlation patterns between soil physicochemical parameters and cyanobacterial biomass during biocrust formation, with the NFB and ESN groups showing the strongest associations with strengthened positive links between NH_4_^+^-N and both Chl-a and microbial biomass.

## 4. Discussion

### 4.1. Potential Analysis of Strains for SynComs Construction in ACCs

The EPS-producing, siderophore-producing, and nitrogen-fixing bacteria that were screened in this work to construct SynComs show significant functional potential in ACCs formation and soil nutrient transformation. EPS, secreted by microorganisms, serves as the core biocementing agents for soil particle aggregation in BSCs formation. In addition to directly improving soil structural stability through ionic and hydrogen bonding interactions with soil particles [48,49], EPS also increases water-holding capacity through pore-filling effects, which are important mechanisms behind the creation of early crusts [50]. In this study, three EPS-producing bacterial strains isolated from natural cyanobacterial crusts showed high EPS production capacities, ranging from 265 to 769 mg·L^−1^. This is significantly higher than the EPS yield (75.4 mg·L^−1^) of previously reported strains with demonstrated efficacy in soil stabilization [51]. These findings imply that our isolated strains have strong biocementing abilities, which could hasten the production of ACCs by promoting soil particle aggregation and offering a more conducive environment for cyanobacterial colonization. Strains screened in this study exhibited siderophore production rates of 55.4~63.4%, which is much higher than the 30% rate reported for strains that improve soil fertility in previous studies [29,52]. In alkaline soils with poor Fe^3+^ solubility, high siderophore activity increases iron bioavailability. By absorbing Fe^3+^-siderophore complexes, non-siderophore-producing microbes can obtain iron, a “public goods” impact that improves the stability of the community as a whole [53,54]. Additionally, siderophores have the ability to chelate additional trace metals, such as Mo, Mn, and Co [55], which relieves a number of elemental constraints that limit microbial growth in hard soils. The four nitrogen-fixing bacteria examined in this study have nitrogenase contents ranging from 333 to 391 pg·mL^−1^, suggesting a strong potential for nitrogen transformation. Interestingly, these strains had a unidirectional promoting impact on bacteria that produced siderophores and EPS. We hypothesize that this could happen through the release of particular metabolites, such as vitamins, amino acids, or organic acids, which could promote the growth of more useful microorganisms in the co-culture system [56,57]. When taken as a whole, these comparisons highlight the competitiveness of our selected strains.

### 4.2. Mechanism Insights into SynComs-Mediated ACCs

The results of microbial biomass and Chl-a content measurements indicated that the addition of mixed bacteria, nitrogen-fixing bacteria, and EPS-producing bacteria markedly enhanced the growth of both cyanobacteria and bacteria, accelerated the formation of ACCs, with the mixed bacteria showing the strongest promotion. Although siderophore-producing bacteria have a minor stimulatory effect on cyanobacterial development, this shows that iron availability is not the main obstacle during initial colonization. The optimized performance of the mixed SynComs indicates a synergistic interaction of microorganisms with different roles. The growth of biocrusts necessitates the simultaneous resolution of problems like micro-environmental stability and nutritional balance [6,58,59], which single-function microorganisms are unable to adequately handle. This synergy supports the multi-factor regulatory theory of biocrust formation.

The stimulating effects of EPS-producing and nitrogen-fixing bacteria on the formation of ACCs were found to vary significantly over time. These differences may be closely associated with the microenvironmental conditions in different formation stages of the biocrusts and the interactions among microbial communities. EPS-producing bacteria appear to play a foundational role by rapidly secreting EPS that stabilize soil particles. EPS facilitates the formation of soil aggregates and enhances soil water retention and stability [50,60], thereby creating a more favorable microhabitat for cyanobacterial colonization. On the other hand, nitrogen-fixing bacteria exhibit delayed but increasingly significant contributions. Their early growth may initially compete with cyanobacteria for limited carbon and energy resources, explaining the transient suppression of Chl-a accumulation. However, as photosynthetically fixed carbon accumulates over time, nitrogen-fixing bacteria become metabolically active, entering a reciprocal relationship.

Additionally, after 24 days of cultivation, the treatment group with nitrogen-fixing bacteria had lower urease activity and ammonium nitrogen content than the other treatment groups. Recent research by Heredia-Velasquez et al. [61] indicates that the form of nitrogen exchange between *Microcoleus vaginatus* and heterotrophic nitrogen-fixing bacteria was urea rather than ammonium nitrogen. Furthermore, the nitrogen-fixing bacteria employed in this work is a member of the same genus as the heterotrophic *Bacillus* sp. O64 used in that investigation. Therefore, we hypothesize that *Microcoleus vaginatus* receives urea from the heterotrophic nitrogen-fixing bacteria, which suppresses urease activity and lowers the build-up of ammonium nitrogen, a degradation product of urea.

Regarding the observed decline in SOM following inoculation, we propose that this reflects active microbial mineralization and respiratory consumption of organic compounds released from dying cells during the adaptation period. All treatments showed a significant drop in microbial biomass at the start of inoculation, along with a concurrent rise in SOM content. This pattern is consistent with other research showing that during the initial inoculation phase, cyanobacteria experience adaptive mortality in response to environmental stress [62]. The increase in SOM likely provides an initial carbon source for subsequent microbial colonization. This pool is subsequently utilized by surviving heterotrophs, which reduces net SOM reduction despite ongoing biomass input. Thus, the transient drop in SOM may represent a dynamic phase of microbial community restructuring.

It is undeniable that the reciprocal balance observed after 24 days of cultivation may reflect short-term laboratory outcomes. In natural environments, biocrusts undergo seasonal fluctuations in temperature, moisture, and nutrient availability, which could disrupt or modify the mutualistic relationships observed here. Therefore, while our SynCom shows promise for rapid ACCs establishment, long-term stability will depend on its adaptability in field applications.

## 5. Conclusions

This study shows that SynComs can effectively promote the formation of ACCs. Among them, the most pronounced facilitative effect was observed with the combined SynComs treatment incorporating all three functional groups, followed by the groups with EPS-producing or nitrogen-fixing bacteria. The underlying mechanism involves a stage-specific functional relay: During the early phase (≤17 days), EPS-producing bacteria enhance soil microaggregate formation and establish a protective physical layer through EPS secretion. This alleviates environmental stress on cyanobacteria in bare sand habitats and markedly elevating cyanobacterial biomass. Subsequently, in the later phase (17–24 days), nitrogen-fixing bacteria form a stable mutualism with cyanobacteria, establishing a nutrient-complementary relationship that drives substantial cyanobacterial biomass enhancement. Although siderophore-producing bacteria failed to enhance cyanobacterial biomass, they enhanced microbial biomass by increasing the bioavailability of trace elements. SynComs transformed the weak correlations observed in the control into a strong positive correlation between NH_4_^+^-N and both Chl-a and microbial biomass. Altogether, our results confirm that the functional SynComs design strategy successfully increases ACCs formation efficiency, providing a new and controllable microbial technology paradigm for the restoration of desert ecosystems.

## Data Availability

The data presented in this study are openly available in [NCBI] at [https://submit.ncbi.nlm.nih.gov/subs/?search=SUB15786987 (accessed on 21 November 2025)], reference number [SUB15786987].

## Supplementary Materials

The following supporting information can be downloaded at https://www.mdpi.com/article/10.3390/microorganisms14010243/s1. Figure S1: Growth curves of highly active EPS-producing bacteria (a), siderophore-producing bacteria (b), and nitrogen-fixing bacteria (c); Figure S2: Photos of plate confrontation between strains with the same function; Figure S3: In the plate confrontation experiment, the colony diameter (mm) between strains with the same function. (a) EPS-producing bacteria, (b) nitrogen-fixing bacteria, (c) siderophore-producing bacteria; Figure S4: Photos of plate confrontation between different functional strains; Figure S5: Diameter (mm) of strain N13 when co-cultured with other functional strains in plate confrontation assay.
