# Supplementary material for: Synthetic Microbial Communities Enhance Artificial Cyanobacterial Crusts Formation via Spatiotemporal Synergy
Source: Microorganisms. 2026 Jan 21;14(1):243. doi: 10.3390/microorganisms14010243 (PMC12844418; doi:10.3390/microorganisms14010243)
Supplement: Supplementary file 1 [file microorganisms-14-00243-s001.zip › microorganisms-4030210-supplementary-proof.pdf]

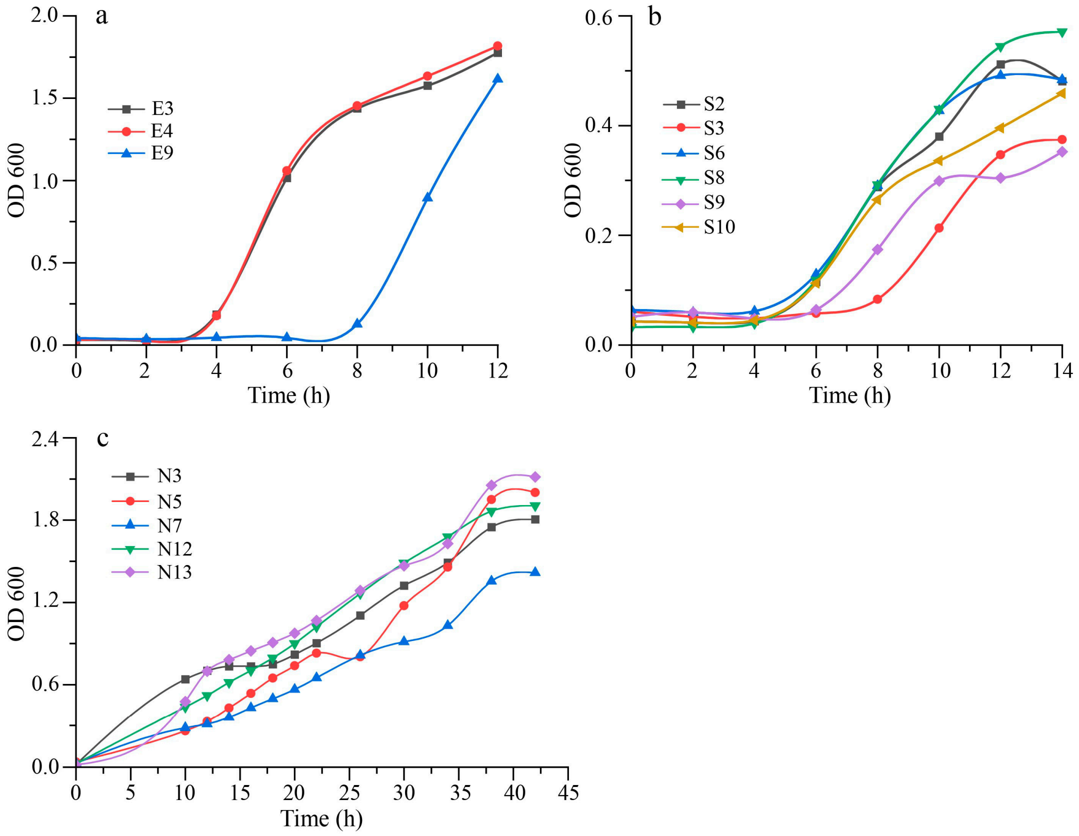

Figure S1 Growth curves of highly active EPS-producing bacteria (a), siderophore-producing bacteria (b), and nitrogen-fixing bacteria (c).

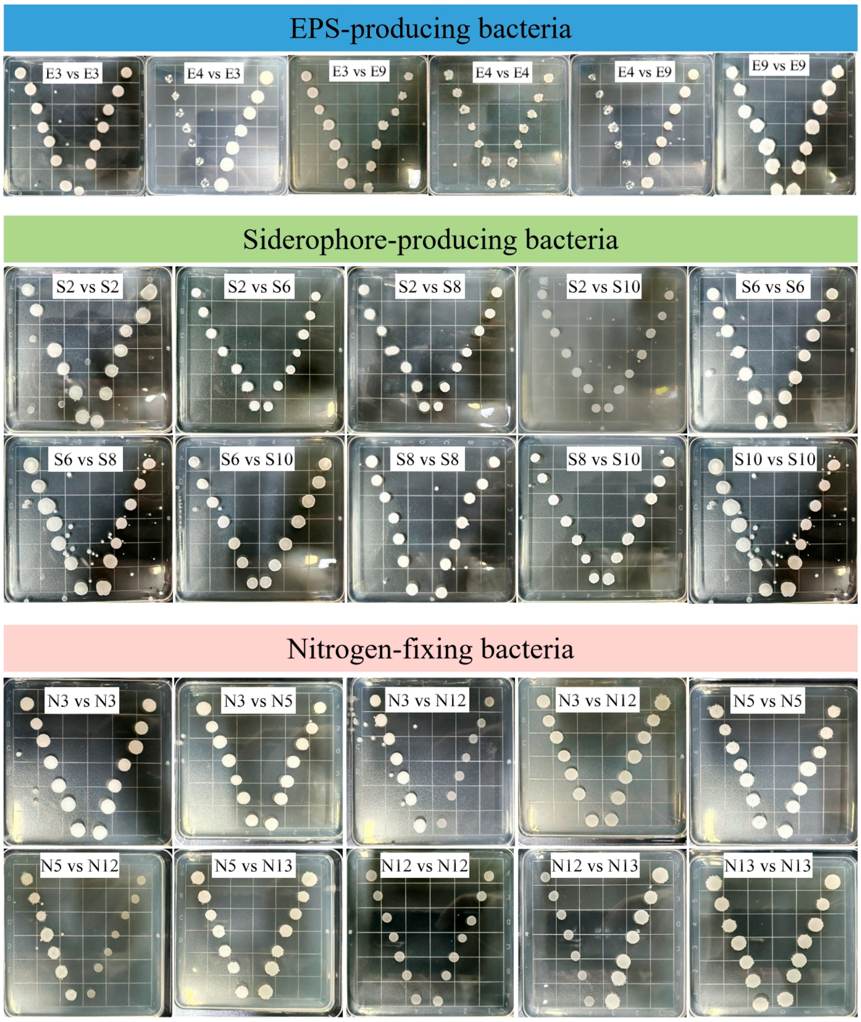

Figure S2 Photos of plate confrontation between strains with the same function

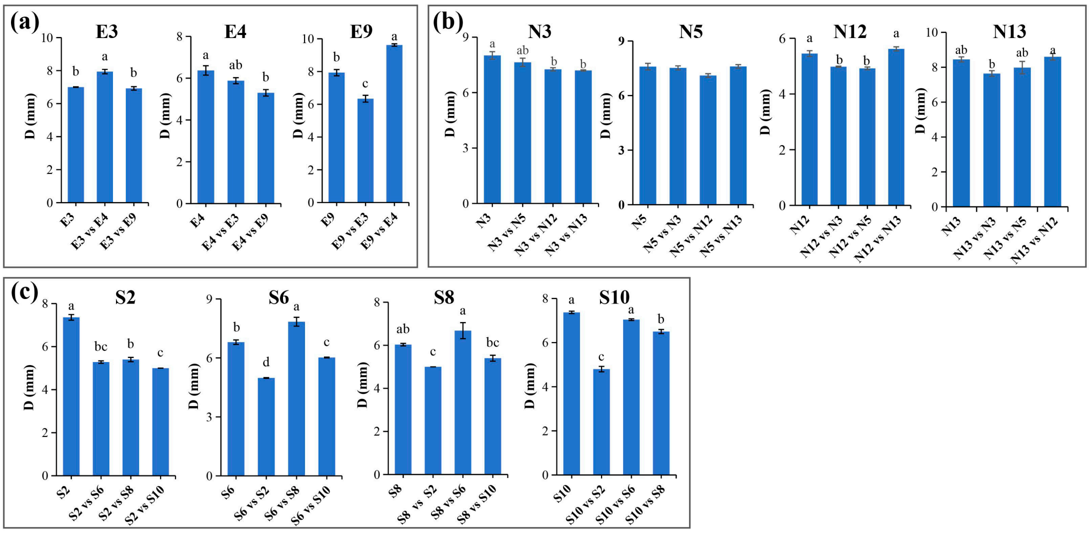

Figure S3 In the plate confrontation experiment, the colony diameter (mm) between strains with the same function. (a) EPS-producing bacteria, (b) Nitrogen-fixing bacteria, (c) Siderophore-producing bacteria.

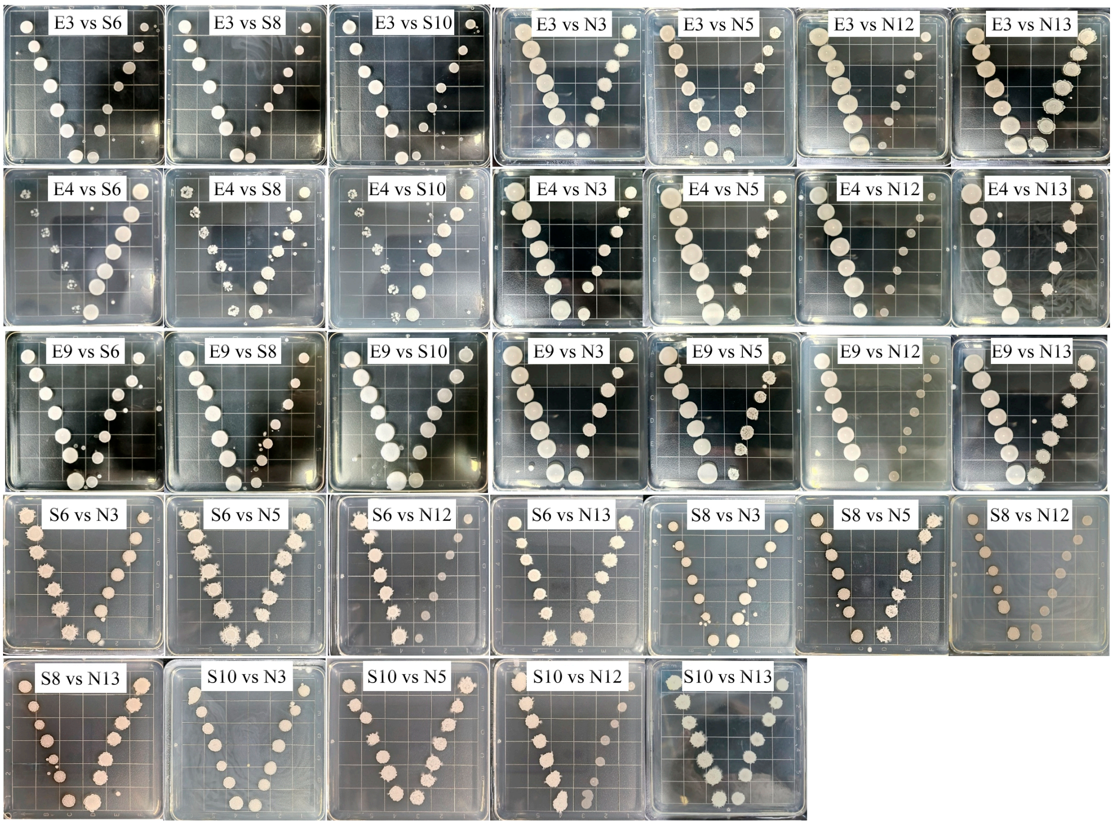

Figure S4 Photos of plate confrontation between different functional strains.

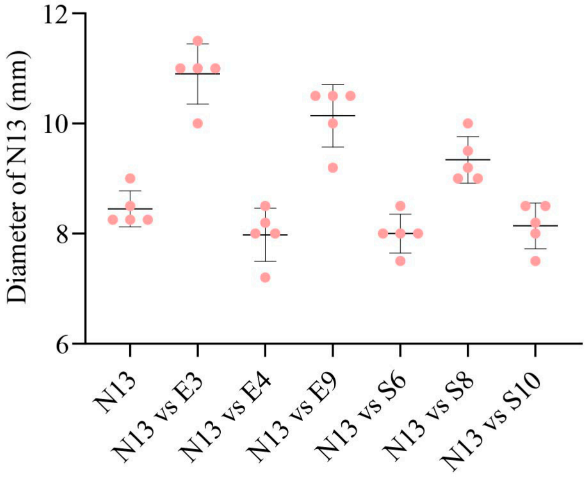

Figure S5 Diameter (mm) of strain N13 when co-cultured with other functional strains in plate confrontation assay.
